# Projections in Various Scenarios and the Impact of Economy, Population, and Technology for Regional Emission Peak and Carbon Neutrality in China

**DOI:** 10.3390/ijerph191912126

**Published:** 2022-09-25

**Authors:** Song Wang, Yixiao Wang, Chenxin Zhou, Xueli Wang

**Affiliations:** 1 School of Business Administration, Northeastern University, Shenyang 110167, China; 2 The Key Laboratory of Carbon Neutralization and Land Space Optimization, Nanjing University, Nanjing 210023, China; 3 Economics and Management School, Wuhan University, Wuhan 430072, China

**Keywords:** emission peak, carbon neutrality, forecasting, influencing mechanisms, China

## Abstract

Owing to the surge in greenhouse gas emissions, climate change is attracting increasing attention worldwide. As the world’s largest carbon emitter, the achievement of emission peak and carbon neutrality by China is seen as a milestone in the global response to the threat. By setting different “emission peak” and “carbon neutrality” paths, this study compares the different pathways taken by China towards regional emission reduction to illustrate China’s possible contribution to global emission reduction, and analyzes the role that China’s economy, population, and technology need to play in this process through the Stochastic Impacts by Regression on Population, Affluence, and Technology model. In terms of path setting, based on actual carbon emissions in various regions from 2000 to 2019 and grid data on land use from 2000 to 2020, the model simulates three emission peak paths to 2030 and two carbon neutrality paths to 2060, thus setting six possible carbon emission trends from 2000 to 2060 in different regions. It is found that the higher the unity of policy objectives at the emission peak stage, the lower the heterogeneity of the inter-regional carbon emission trends. In the carbon neutrality stage, the carbon emissions in the unconstrained symmetrical extension decline state scenario causes the greatest environmental harm. Certain regions must shoulder heavier responsibilities in the realization of carbon neutrality. The economic development level can lead to a rise in carbon emissions at the emission peak stage and inhibit it at the carbon neutrality stage. Furthermore, the dual effects of population scale and its quality level will increase carbon emissions at the emission peak stage and decrease it at the carbon neutrality stage. There will be a time lag between the output of science and technology innovation and its industrialization, while green innovation is a key factor in carbon neutrality. Based on the results, this study puts forward policy suggestions from a macro perspective to better realize China’s carbon emission goals.

## 1. Introduction

Global climate change is currently the most important environmental concern in the world [1]. In particular, anthropogenic greenhouse gases can cause major harmful changes in climate in the 21st century [2]. They also have an unprecedented short- and long-term impact on the environment, society, and economy. Carbon dioxide (CO_2_) is the most important component of anthropogenic greenhouse gases [3]. Adopting active emission reduction and carbon removal policies has received international political consensus to deal with climate change [4]. Limiting global warming below 2 °C and striving to reach the global temperature control target of 1.5 °C are expected to avoid the irreversible negative impact of climate change on human society and natural ecosystems. However, this process requires joint efforts from all countries in terms of controlling carbon emissions. On 16 September 2020, Ursula von der Leyen, the President of the European Union, delivered the “State of the Union”, and announced that by 2050, Europe will become the world’s first carbon-neutral continent. By the end of 2020, a total of 44 countries and economies announced their carbon neutrality goals. On his first day in office, Joseph Robinette Biden, the new US President, signed an executive order to return the country to the Paris Agreement and reach carbon neutrality by 2050.

Compared with developed countries, the energy demand of developing countries, such as China, has increased sharply with economic growth [5]. In the past decade, China’s carbon emissions from fuel combustion have always ranked first [6]. Therefore, in the process of carbon removal, China will face complex challenges such as large total carbon emissions, short time of carbon emission reduction, difficult economic transformation, and cumbersome transformation of energy systems. To slow down global warming, China is duty bound to undertake emission reduction and commit itself to reaching the peak of carbon emissions in 2030 and carbon neutrality in 2060 [7].

Recently, China has been shifting from the traditional economic development model of “high investment, high consumption, and high pollution” to a sustainable development model to build an ecological civilization and achieve high-quality economic growth, through a low-carbon development path with innovative development modes [8]. However, as the world’s largest developing country, China needs to take broader and stronger emission reduction actions over a shorter period to achieve carbon neutrality by 2060, which necessitates the cooperation of the populace and each region. The Chinese government has made it clear that emission peaking and carbon neutrality are not only macro-national objectives but also specific, local ones. All regions should achieve emission peak and carbon neutrality at the established time points.

China has a vast territory and there are great differences in natural endowment, economic development level, social development, and science and technology (S&T) innovation capacity among regions. Therefore, determining an effective low-carbon development path based on the actual development of the various regions to achieve emission peak and carbon neutrality in all regions within the expected time demands serious attention and thought.

This study makes regional-level projections and identifies the influencing mechanisms of China’s carbon emissions. Specifically, we focus on building divergent carbon emission change modes at the regional level, composed of two carbon emission thresholds and their intermediate levels, and combined with two carbon neutrality paths of the symmetrical extension decline state and the uniform decline state. The impact paths of the six different carbon emission scenarios differ. Since a total carbon emission control mechanism based on the regional objectives of emission peak and carbon neutrality has not been fully established, this study simulates the threshold and possible trend of carbon emissions from 2020 to 2060 at the regional level to determine the peak value of carbon emission in each region and the total carbon emission over the entire period. Thus, it explores China’s overall contribution to carbon emissions. Based on the path analysis results under the different scenarios, this study also puts forward phasic and regional policy suggestions for the Chinese central government and local governments in multifarious aspects, including carbon emission control, economic development level, population scale, and S&T innovation. These measures can help local governments formulate reasonable tailored emission reduction plans.

This study focuses on three parts: regional emission peak scenario with projection; regional carbon neutrality wish scenario with projection; and carbon emission influencing factor analysis. The remainder of the paper is organized as follows. First, it reviews the literature on three key aspects: carbon emission; emission peak with carbon neutrality; and factors affecting carbon emissions to clarify the research contents and research methods of extant studies. Second, it measures the carbon emissions of various regions in China and simulates their emission peak scenarios according to three preset scenarios (the unconstrained state, ideal state, and average state scenarios) based on the “2006 IPCC Guidelines for National Greenhouse Gas Inventories”. Third, it measures and predicts the carbon sink of each region in China based on land use and simulates six scenarios of carbon neutrality in each region according to the preset carbon removal scenario from 2020 to 2060—unconstrained symmetrical extension decline state, ideal symmetrical extension decline state, average symmetrical extension decline state, unconstrained uniform decline state, ideal uniform decline state, average uniform decline state. Next, it constructs a model of Stochastic Impacts by Regression on Population, Affluence, and Technology (STIRPAT) to explore the influence and effects of the economic development level, population scale, and S&T innovation on environmental impact during the measurement (2000–2019), emission peak (2020–2030), and carbon neutrality periods (2031–2060). Finally, based on the empirical results, this study puts forward policy planning for China’s central government and its local governments to help achieve emission peak and carbon neutrality and proposes a path of low-carbon development based on three impact dimensions.

## 2. Literature Review

### 2.1. Research on Carbon Emission

Climate change has been identified by scientists, policymakers, and even the public as one of the two most important problems to be solved worldwide (the other is the shortage of water resources) and has a considerable impact on economic and social development [9]. As the world’s largest coal consumer and coal-derived power generation country during its economic and industrial development, China’s uncontrolled consumption of fossil fuels has led to large greenhouse gas emissions [10]. Climate problems not only worsen air quality and endanger human health [11] but also affect macroeconomic growth [12]. Greenhouse gases include water vapors (H_2_O), carbon dioxide (CO_2_), nitrous oxide (N_2_O), freon, and methane (CH_4_). Among them, CH_4_ accounts for approximately 35% of mixed greenhouse gases and is the second largest contributor to global warming [13]. Over the past three decades, scholars have realized the importance of the qualitative assessment of CH_4_ emission inventory and have conducted studies on CH_4_ emissions worldwide [14]. This research comprised the exploration of CH_4_ emissions of different land use types, such as paddy fields and wetlands, and the exploration of specific conditions in different regions, such as the Makran continental margin [15], Latin America [16], and China [17]. However, the short-term climate forces of greenhouse gases, such as CH_4_, have a shorter lifetime in the atmosphere compared to CO_2_ and are generally considered to only have a temporary impact on the climate system [18]. Among these anthropogenic greenhouse gases, CO_2_ accounts for nearly three-quarters of all greenhouse gas emissions, and is regarded as the most important component that mitigates global warming [19].

In the natural sciences, scholars have focused on emerging technologies, such as carbon capture and storage (CCS) [20], while in the social sciences, they have emphasized the measurement and estimation of carbon emissions [21] and the establishment of corresponding social systems, such as carbon taxes [22] and carbon trading [23], and scientific carbon forecasting [24]. Currently, there are no monitoring data that can be directly applied to actual carbon emissions in China, which is why it needs to be measured through indirect methods. Carbon emissions can be measured using various methods under diverse circumstances. For instance, the Input–Output Analysis (IOA) model is used to explore carbon emissions in some regions [25]; The Multi-Regional Input–Output (MRIO) model based on interregional commodity flow data is an effective tool for cross-regional emission research [26]; The Life Cycle Assessment (LCA) model is used to analyze the carbon emissions of a single product or process; and the Denitrification–Decomposition (DNDC) model has been developed and applied to measure agricultural carbon emissions [27]. The calculation of carbon emissions based on the “IPCC Guidelines for National Greenhouse Gas Inventories” and the law of mass conservation is a more universal carbon measurement method [28]. Based on these varied calculation methods, the measurement of regional carbon emissions is also widely carried out, including in specific economic development regions, such as BRICS (Brazil, Russia, India, China, South Africa) [29] and APEC [30], as well as in countries [31], counties [32], cities [33] and enterprises [34]. For China, the measurement of carbon emissions also covers many provinces, such as Fujian [35], Henan [36], Inner Mongolia [37], Anhui [38] and so on.

### 2.2. Research on Emission Peak and Carbon Neutrality

China is facing the dual pressure of air pollution and climate change mitigation [39]. Seeking harmonious development between humans and nature, and actively exploring ecological civilization construction adapted to national conditions, have become the current focus [40]. Under the Paris Agreement, China has committed to reach the peak of carbon emissions by 2030 and achieve carbon neutrality by 2060. However, achieving these goals with minimum economic and social costs is a problem worth analyzing [41]. Regarding the goal of reaching the emission peak in 2030, several studies have investigated the peak of China’s carbon emissions from different perspectives, including the conditions for reaching the peak. For example, it was considered that in order to reach the emission peak, the decline rate of carbon intensity should be higher than the economic growth rate [42]. It was found that the change in total factor productivity plays an important role in the realization of the emission peak [43]. The exploration of emission peak time based on different influencing factors, such as urbanization [44] and economic structure [45], was also investigated. Moreover, based on divergent peaking scenarios, increased attention has been paid to the analysis of emission peaking in construction [46], service [47], power and heating [48], household [49], transportation [50], and other sectors.

Owing to the carbon sink problem, carbon neutrality is more complex than the emission peak, thus resulting in fewer studies in this area; however, research on carbon sinks provides interesting findings for scholars. Global research focuses on the protection of the ecological environment as terrestrial ecosystems are generally considered to provide a powerful means to mitigate climate change [51]. Coastal ecosystems covered by mangroves, seagrass meadows, and tidal swamps also have high carbon sequestration capacity; therefore, the role of blue carbon in mitigating climate change is considered very important [52]. Whether in terrestrial or coastal ecosystems, the carbon sink of the ecological environment is key to achieving carbon neutrality. Research also focused on the measurement and projection of carbon sinks for different land use types. When dealing with ecosystem carbon sequestration, some studies examined the interconnection and integrity of each ecosystem and the creation of complex dynamic models, such as the Yale Interactive terrestrial Biosphere (YITBs) model [53] and Cellular Automata (CA) model [54]. Most studies use static carbon sink coefficients for varying land types [55]. Although more than 60% of ecosystems have been degraded by human activities, the change of soil organic carbon (SOC) still takes a long time, even under the influence of human activities [56]. Therefore, most studies assume that human activities have no short-term impact on ecological carbon absorption capacity. Relevant research has obtained the land use data from the inversion results of satellite monitoring [57]. The land cover area and static carbon sink coefficient jointly determine the carbon sink scale of the ecosystem.

### 2.3. Research on Carbon Emissions Determinants

Recently, with the gradual increase in the study on the impact of climate change on various human activities, scholars have also begun to pay attention to the factors influencing climate change [58]. Although the factors affecting the growth of carbon emissions are complex and diverse, the economy, population, and S&T innovation are the three factors primarily considered by scholars [59]. The impact of economic growth on the environment is regarded as one of the foundations of economic research, but the academic community has different opinions on the relationship between the two [60]. The classic hypothesis on this aspect is the Environmental Kuznets Curve (EKC) hypothesis proposed by Grossman and Krueger, that is, the economic level and carbon emissions have a relationship in the form of an inverted U-shape [61]. Some studies verify this hypothesis by setting different research areas, such as Malaysia [62], Brazil, China, India and Indonesia [63], 15 countries in the Middle East and North Africa and Pakistan [64], or by incorporating various influencing factors, such as ecological footprint [65], and foreign direct investment (FDI) [66].

Most of these studies believe that the environmental deterioration caused by economic growth is short term, and environmental problems will be solved gradually with further economic growth [67]. Others emphasize the decoupling of economic growth and carbon emissions to control environmental degradation [68]. However, there is no evidence that the decoupling is endogenous; hence, a strong emission reduction policy support and adaptation to economic growth and sustainable development are required [69]. The associated population attributes are also highly related to the changes in carbon emissions. The extant research mainly focuses on the factors of population structure change, such as population growth and population size [70]. Population growth is considered a key factor to the change in the residents’ carbon emissions [71]. Its main forms are natural population growth and population migration. The former directly leads to an increase in carbon emissions, while the latter spatially reconstructs the scale of carbon emissions by causing changes in production and consumption activities [72]. The impact of the two forms of population growth on carbon emissions is the change in population structure and its related parameters. These fluctuations are the key to the subsequent change in carbon emissions growth rate. For example, population aging caused by natural population growth can offset carbon emissions [73], while urbanization caused by population migration can promote carbon emissions [74]. Environment and innovation are the two most important themes in global development [75], while the innovation is the key factor of social change [76], but S&T innovation is a double-edged sword for ecological protection. On the one hand, it may cause problems to the environment and urban development, such as air pollution [77]. On the other hand, the negative impact of innovation may only be short term and innovation can inhibit carbon emissions in the long term [78]. Currently, several studies have begun to pay attention to the innovation related to environmental protection, that is, green innovation. It can help promote global energy conservation and carbon emissions reduction to a large extent, and be the driving force to achieve both economic growth and green low-carbon transformation [79].

While the scope of research on the influencing factors of carbon emissions continues to expand, the methods used to explore its effects also increases. Some studies have used the factor decomposition method to analyze the relationship between indicators and their driving factors to determine the impact of the latter. Research has also analyzed agricultural carbon emissions based on the exponential decomposition method [80], industrial carbon emissions based on Structural Decomposition Analysis (SDA) model [81], and transportation industry carbon emissions [82] and power sector carbon emissions [83] based on the Log-Mean Divisia Index (LMDI) model. However, this type of research lacks a unified framework for the influencing factors, and the STIRPAT model framework effectively solves this problem. The STIRPAT framework is based on the IPAT (Impact = Population × Affluence × Technology) model proposed by Ehrlich and Holdren [84], and proposed by Dietz and Rosa [85] through the regression of population, wealth and technology, and the random estimation of environmental pressure, which is used to quantitatively analyze the impact of human factors on environmental pressure. Currently, it is widely used as the starting point to estimate carbon emissions under different assumptions [86]. Moreover, some studies have carried out inverse estimations, such as analyzing the elasticity coefficient of the influencing factors of carbon emissions in a single region [87]. On account of the scalability of the STIRPAT model, studies have also considered the urbanization level, urban employment level, industrialization level [88], total nuclear energy, alternative energy and total fossil energy [89].

We can see that previous studies have mostly focused on the measurement of carbon emissions in the past before carrying out relevant research on the carbon peak in sub-sectors based on said measurements. However, these studies seldom consider the realization of carbon neutrality goals and are lacking from a macro perspective. Although various methods and models have been used in past research on the influencing factors of carbon emissions, these studies are mostly a summary of past experiences. They rarely predict anything about the future role of influencing factors. Compared to the existing studies, the novelty of this paper is as follows: (1) taking China’s provincial administrative regions as the research objects, this paper analyzes the vision of China’s regional carbon peak and carbon neutrality from a macro perspective; (2) By setting different scenarios, the high (low) threshold of regional carbon peak is simulated, and the possible range of carbon emissions is given; (3) Based on the simulation of different carbon peaks, the situation of regional carbon neutralization is simulated, which comprehensively reflects the total carbon emissions under the vision of carbon peak and carbon neutrality; (4) The future development of China’s regional economy, population, and S&T are simulated to analyze the role of various influencing factors in different periods.

## 3. Regional Emission Peak

### 3.1. Measurement of the Regional Emission Peak

Quantifying the emissions of a country or region is the first step towards reducing greenhouse gas emissions [90]. This study selects regional final energy consumption data to measure regional carbon emissions, with data from the “China Energy Statistics Yearbook”. The Coronavirus Pandemic (COVID-19) has brought a huge impact to China after 2019 [91]. Considering the significant change in carbon emissions caused by COVID-19 [92] and its impact on subsequent projections, the measurement period comprises the two decades (2000–2019). The potential carbon emissions for each province were calculated according to the carbon emission calculation guide methods and parameters in the “IPCC Guidelines for National Greenhouse Gas Inventories” issued by the Intergovernmental Panel on Climate Change (IPCC) in 2006. The specific calculation method is as follows:

(1) $$CE=\sum_{j=1}^{n}E_{j}\times NCV_{j}\times CEF_{j}\times COF\times \frac{44}{12}$$

where *CE* is the sum of the CO_2_ emissions from various energy sources. According to the “China Energy Statistics Yearbook” energy is divided into 10 categories—coal, coke, crude oil, gasoline, kerosene, diesel, fuel oil, liquefied petroleum gas, natural gas, and electricity. *E_j_* is the consumption of the *j*-th energy type, *NCV_j_* is the average low calorific value of the *j*-th energy type, *CEF_j_* is the carbon content per unit of calorific value of the *j*-th energy type, *COF* is the carbon oxidation factor (usually one, according to IPCC (2006)), and $\frac{44}{12}$ is the molecular weight ratio of CO_2_ to Carbon (C).

**Figure 1:**
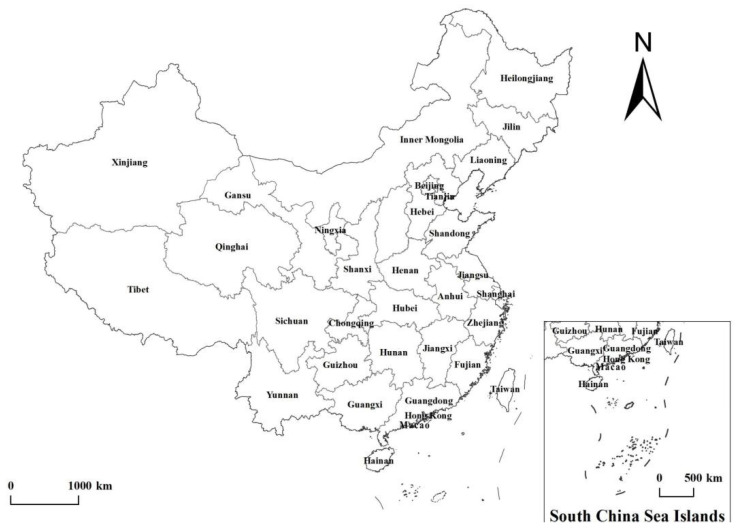
Geographical location of the research objects.

The regions selected in this paper are China’s provincial administrative units, and the geographical location of each provincial administrative region is shown in Figure 1. For the sake of data integrity and statistical quality, the research objects excluded Tibet, Hong Kong, Macao, and Taiwan.

### 3.2. Estimation of the Regional Emission Peak

#### 3.2.1. Scenario Setting of Emission Peak

Based on the measured regional actual carbon emissions from 2000 to 2019, and without considering the upheavals in natural factors, such as major disasters, the impact of human factors, including conflict and war, the basic constraints of revolutionary S&T innovation, and the application of new energy, we set the unconstrained state, ideal state, and average state scenarios. The carbon emission forecast assumptions for each scenario are as follows.

**Scenario 1: Unconstrained state**

**Figure 2:**
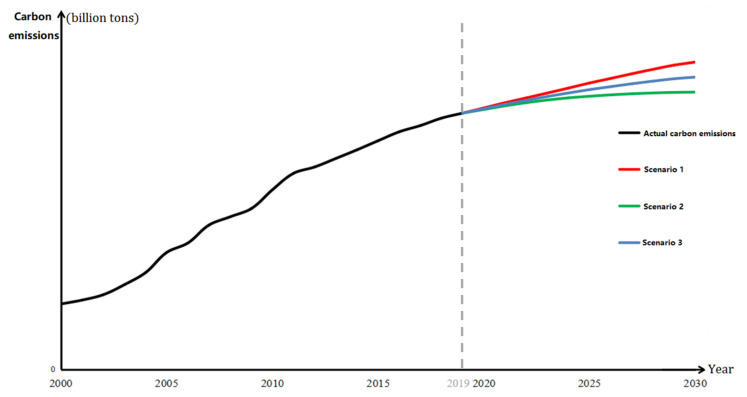
Schematic diagram of different scenario settings for emission peak projection.

Without policy intervention, regional carbon emissions are considered to increase naturally according to the historical data trend, and the government will not take measures to limit the rate of carbon emissions (as shown by the red solid line in Figure 2). Although this scenario is not in line with the reality, the predicted value of carbon emission here is likely to be the maximum boundary of carbon emission in the actual setting, which means it will be difficult to achieve carbon neutrality wish after 2030.

**Scenario 2: Ideal state**

Ideally, the government’s emission reduction policy is fully effective and the regional carbon emissions will gradually decrease at an annual growth rate, reaching zero in 2030. In such a scenario, the projection is in line with the realization of the emission peak and carbon neutrality goals, as it not only simulates the minimum carbon emission in each region, but also avoids huge fluctuations in regional carbon emissions. Without considering the governments’ use of coercive measures at the expense of the economy, the predicted value of carbon emission in this scenario is likely to be the minimum boundary of carbon emission in reality, which makes it the easiest emission peak scenario in achievingcarbon neutrality wish after 2030.

**Scenario 3: Average state**

Based on scenarios 1 and 2, the possible maximum value (predicted value under the unconstrained state) and possible minimum values (predicted value under the ideal state) of carbon emission in each region are averaged. The average carbon emission is the average of two extreme values, which may be closer to reality.

#### 3.2.2. Estimation Method of Emission Peak Scenarios

In scenario 1, given that the annual growth rate of regional carbon emissions generally presents the distribution characteristics of “more in the middle while less on both sides”, the kernel density estimation method based on the “normal” kernel function is applied to the annual growth rate of regional carbon emissions from 2001 to 2019. The change step is adjusted with the fit degree as standard with 106 times the simulation by the Monte Carlo method, and the median of the sequence value represents the centralized data trend. Considering the outliers in the growth rate for individual years, the data should be updated annually; that is, the outlier values should be eliminated for each growth rate calculation round, while the number of known data in each round is the same. Through the predicted annual growth rate, the carbon emissions under unconstrained state were calculated on a year-by-year basis.

In Scenario 2, in view of the fact that the historical data of the annual growth rate of regional carbon emissions are converging on both sides with a specific year as the boundary, and the annual growth rate fluctuates above a specific level before a specific year, while it is opposite after a specific year. Based on these features, the growth rate in 2020 is predicted using the corresponding annual growth rate of the specific year as the upper limit, while the growth rate of the previous year becomes the upper limit to predict the subsequent years (2021–2030), and the lower limits are all zero. According to the maximum entropy principle, the growth rate for the next year falls in the middle of the upper and lower limits.

In scenario 3, the annual carbon emission forecast value of each region is the average value of scenarios 1 and 2.

### 3.3. Analysis of the Regional Emission Peak

**Figure 3:**
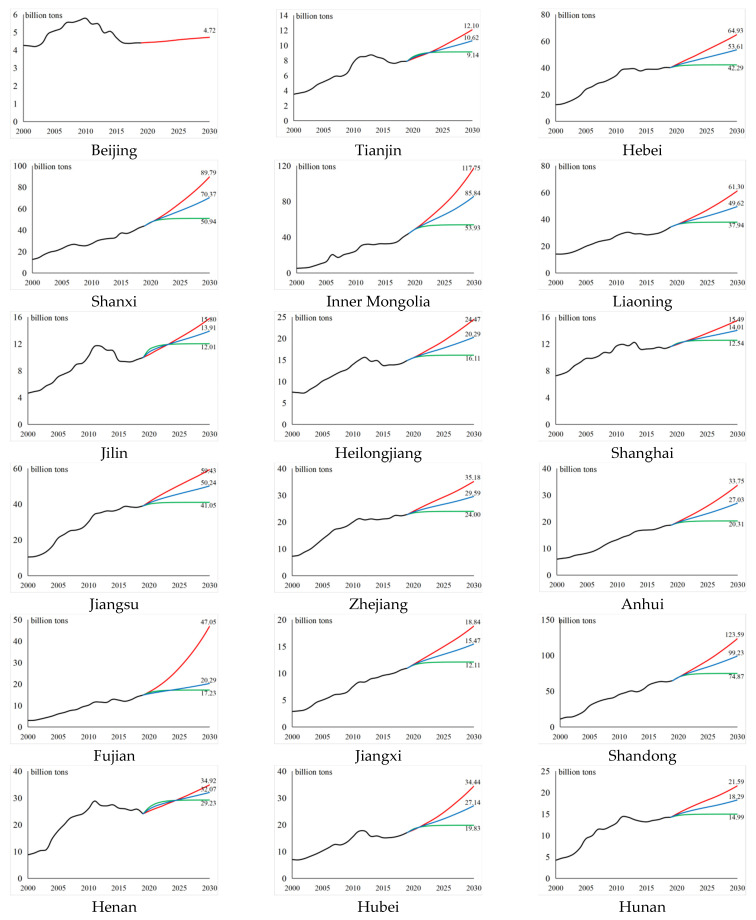

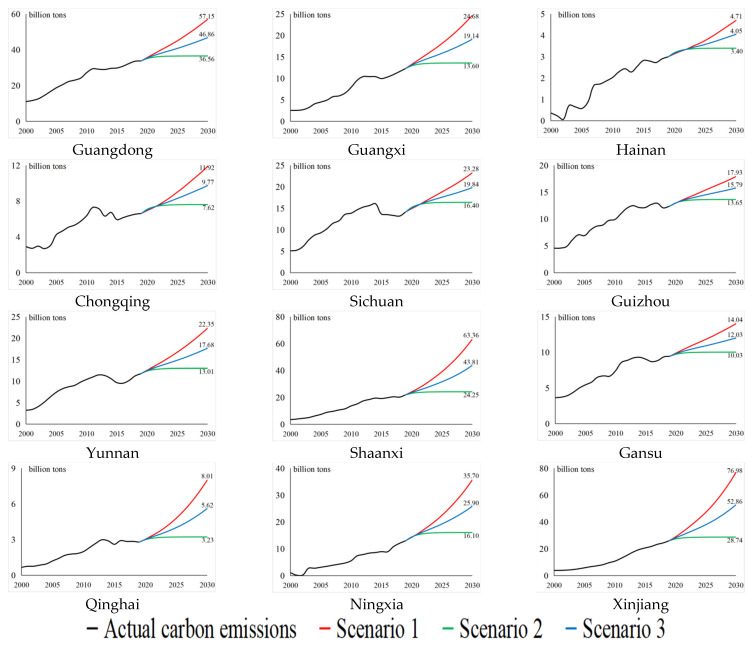
Projection of emission peak in various regions of China.

Based on the possible settings of carbon emissions under the different scenarios, we predicted the carbon emissions for the different regions in China from 2020 to 2030, and the results are shown in Figure 3. (Owing to the large difference in carbon emissions among the regions, to describe the actual carbon emissions from 2000 to 2019 and the emission peak projection from 2020 to 2030 more clearly, the value range of the vertical coordinates for each region is different.) For instance, Beijing reached its emission peak in 2010. Since then, its carbon emissions have generally shown a fluctuating downward trend. Under the unconstrained state, the growth rate of carbon emissions is very low (the annual growth rate is below 1%) or even negative. At the same time, the maximum value over the projection period has been significantly lower than the overall maximum value. It can thus be considered that Beijing reached its emission peak in 2010, which is why other scenarios were not simulated for the city. For the other provinces, the three scenarios were analyzed as follows.

In the unconstrained state scenario, from the growth rate perspective, Inner Mongolia, Fujian, Shaanxi, Qinghai, Ningxia, and Xinjiang showed poor control strength and effect before 2019. If policy control is not applied in the future, these six provinces will have the fastest growth in domestic carbon emissions (with an annual growth rate of around 10%). The annual growth rate of nine provinces, including Jilin and Jiangsu, is below 4.5%. If these regional governments respond actively to the emission peak policy and impose a certain degree of control, it will be easier to achieve the peak of carbon emissions. In terms of range, the range of growth rate between the maximum and minimum emissions is largest (approximately 6%) in all three scenarios, which confirms that, with the development of carbon emissions according to the general historical trend, the difference in carbon emissions between regions will increase annually. In terms of peak value, the peak value of carbon emissions in Shandong is the highest among all the regions, which also makes it difficult to achieve carbon neutrality wish by 2060. Conversely, the peak value of carbon emissions in Hainan is the lowest, at approximately 3.33% of the highest value. The cumulative carbon emissions of all provinces also show heterogeneity, among which Hainan and Qinghai have the lowest cumulative carbon emissions from 2000 to 2030 (below 10 billion tons), while Shandong has the highest cumulative carbon emissions at this stage (approximately 185.9 billion tons).

In the ideal state scenario, from the growth rate perspective, the carbon emission of each region shows saturated growth, and the annual growth rate of each province is below 0.1% from 2026 to 2030, which has led to the goal of reaching the peak under the premise of extremely low carbon emission growth. In terms of range, the difference between regions in the ideal state is smallest. Under the unified goal of reaching the emission peak by 2030, the development trend of carbon emissions in the various provinces will show a convergence. In terms of peak value, the peak value of carbon emissions in Shandong under a fully effective policy is still highest among all the regions. However, based on horizontal comparison, its peak value under this scenario is reduced to 61% under the unconstrained scenario, which reveals that, ideally, the effect of carbon removal is significant. Qinghai is the lowest among all the regions, accounting for only 323 million tons. Similar to the unconstrained scenario, under the ideal state scenario, Hainan and Qinghai have low carbon emissions, and the cumulative carbon emissions of each region will be reduced by 249.8 billion tons compared with the unconstrained state (1807.1 billion tons).

In the average scenario, the annual growth rate of carbon emissions is between the unconstrained state and the ideal state scenarios, the carbon emissions being relatively in line with the real emission range. From the peak value perspective, Shandong is still the province with the highest carbon emissions in 2030 (carbon emissions are 9.923 billion tons) and Qinghai has the lowest emissions, with a significant difference of 9.361 billion tons between them. Under China’s goals of emission peak and carbon neutrality wish, the development of carbon emissions in provincial-level administrative regions may be different in reality, which means that local governments need to respond flexibly. Compared with the unconstrained state scenario, the cumulative value of regional carbon emissions over the past 30 years is approximately 50% of the reduction scale in the ideal state scenario.

Looking at the emission peaks of different regions under different scenarios, we find that the total emissions of China’s mainland (excluding Tibet due to missing data) in the unconstrained, average, and ideal states will reach 117.5 billion tons, 92.8 billion tons, and 68 billion tons, respectively. These figures indicate that the peak value in 2030 will be within [68, 117.5] billion tons. Compared with the unconstrained scenario, optimal control can reduce emissions by nearly half, lower carbon emissions by approximately 50 billion tons by 2030, and decrease carbon emissions by approximately 25 billion tons even under the average state, which means that China’s control over carbon emissions is of great significance globally. The proportions of the eastern, central, and western regions in peak carbon under the unconstrained scenario are 41.32%, 23.28% and 35.40%, respectively, while under optimal control, the proportions become 44.68%, 25.82%, and 29.50%. This result indicates that optimal control greatly restricts the carbon emissions of the western region, by as much as 21.5 billion tons. Additionally, the carbon emissions in the eastern and central regions are also greatly constrained. Only when all regions pursue the local optimization of carbon emissions under the same goal can they reach global optimization for the entire country.

## 4. Regional Carbon Neutrality Wish

### 4.1. Measurement of the Regional Carbon Sink

Regional carbon sequestration was measured based on 1-km land use grid data generated by Landsat TM images, with manual visual interpretation. The data were obtained from the Resources and Environmental Science and Data Center (https://www.resdc.cn, accessed on 30 June 2022) from five different time periods of 2000, 2005, 2010, 2015 and 2020. In this database, land use types are divided into six categories and 25 subcategories, of which urban and rural, industrial and mining, and residential land hardly include carbon sink; therefore, only cultivated land, woodland, grassland, waters, and unused land are discussed. Compared with other carbon sink areas, permanent glacier and snow land in the subcategory corresponding to the water areas has low carbon sink capacity due to the lack of vegetation or microbial respiration in the barren surface moraine areas and the large pores of the surface moraine in the glacier movement. Therefore, it is difficult to retain CO_2_, hence, the carbon sink of permanent glacial and snow land are excluded. Using ArcGIS Pro, the classified and encoded grid data were read and processed into the corresponding number of pixels, and the area of the corresponding land use type were calculated [Area of land use type = number of pixels × single pixel area (1 km^2^)]. The static carbon sink coefficient on different land use types were determined by referring to the extant research on carbon sink capacity for different land use types in high-impact journals. The ecosystem carbon sink model can be estimated as follows:

(2) $$C=\sum_{i=1}^{m}S_{i}\times \alpha_{i}$$

where *S_i_* is the area of the *i*-th land use type, and *α_i_* is the carbon sink coefficient of the *i*-th (*i* = 1, 2, …, *m*) land use type, while the corresponding carbon sink coefficient is shown in Table 1. The regional carbon sink, *C*, is the sum of the different carbon sinks in the region, that is, the sum of the product of *S_i_* and *α_i_*.

### 4.2. Estimation of the Regional Carbon Neutrality Wish

#### 4.2.1. Scenario Setting of Carbon Neutrality Wish

Based on the measured regional actual carbon emissions from 2000 to 2019 and the predicted values under the divergent scenarios from 2020 to 2030, and without considering the upheavals in natural factors, such as major disasters, the impact of human factors, including conflict and war, the basic constraints of revolutionary S&T innovation, and the application of new energy, we set the symmetrical extended decline state, and uniform decline state scenario. Since carbon neutrality means that the carbon emissions and sinks are the same in 2060, instead of setting the carbon emissions to zero, the goal is to reduce the net carbon emissions (i.e., the difference between carbon emissions and carbon sinks) to zero by 2060.

**Scenario 1: Symmetrical extended decline state**

**Figure 4:**
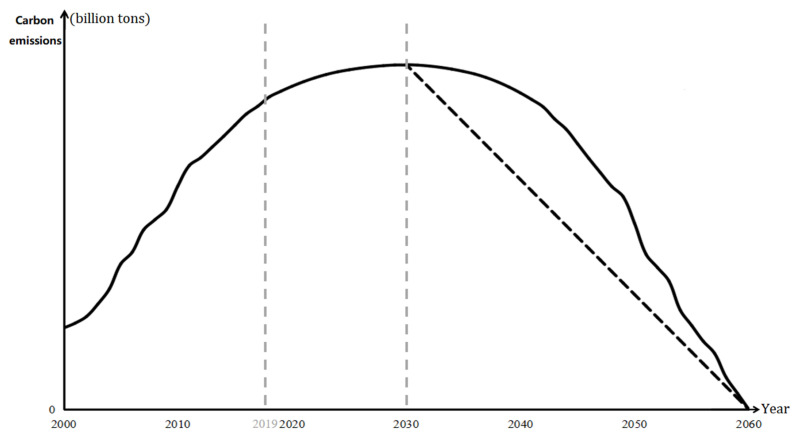
Schematic diagram of different scenario settings for carbon neutrality wish.

**Figure 5:**
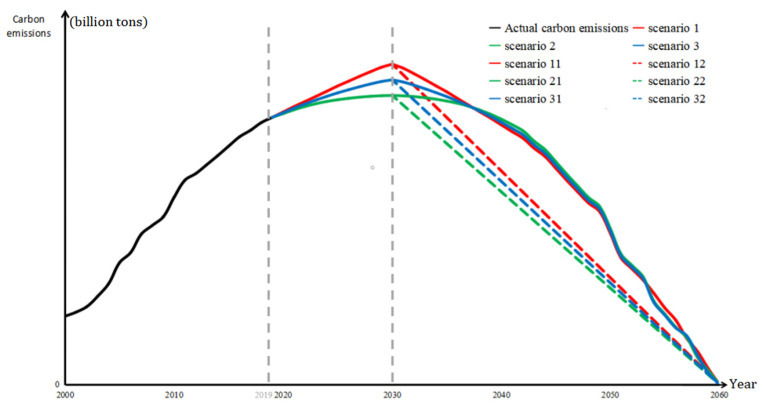
Different scenario combinations of carbon neutrality wish.

The neutralization scenario of symmetrical extension decline state is to estimate the downward trend of the net carbon emission levels from 2031 to 2060 by referring to the historical changes from 2000 to 2030 and taking 2030 as the axis of symmetry, on the basis of the assumption that all regions will reach their emission peak by 2030 under three different scenarios (as shown by the solid line on the right-hand side of 2030 in Figure 4) and each emission peaking scenario has a corresponding carbon neutrality wish scenario—scenarios 11, 21, and 31 achieve carbon neutrality wish by developing in the unconstrained state, ideal state, and average state scenarios to a symmetrical extended decline after the peak year, as shown in Figure 5 and Table 2.

**Scenario 2: Uniform decline state**

The neutralization scenario of uniform decline assumes that all regions will achieve their emission peak by 2030, the quantity level of net carbon emissions will decrease annually, as shown by the dotted line on the right-hand side of 2030 in Figure 4, and all regions will achieve carbon neutrality wish by 2060; that is, the net carbon emissions are zero. Each emission peak scenario also has a corresponding uniform decline scenario—scenarios 12, 22, and 32 develop in the unconstrained state, ideal state, and average state scenarios to achieve emission peaking after 2030, as shown in Figure 5 and Table 2.

#### 4.2.2. Estimation Method of Emission Peak Scenarios

To estimate carbon neutrality wish, it is first necessary to estimate the carbon sink in various periods for each region. Considering that historical data consist of five periods within five-year intervals, it is not suitable to adopt time-series projection or similar methods. Therefore, this study uses a curve fitting and grey model to compare and select the optimal model to predict the carbon sink capacity of each region from 2021 to 2060. The gray combination prediction model is a combination of three gray models, namely, the traditional GM (1,1), the GM (1,1) based on the new information priority principle, and the GM (1,1) based on the optimized background value. On the premise of using the exponential of smoothing ratio analysis data as a model, combined with an error square for optimal model selection, the average relative residual and average pole ratio deviation are used as criteria to judge the goodness of fit. Based on the estimated carbon sequestration, the carbon emissions during the stage of carbon neutrality wish under the divergent scenarios were estimated to render regional carbon emissions equal to carbon sequestration by 2060.

In scenario 1, based on the actual carbon emission level from 2000 to 2019 and the three carbon neutrality wish scenarios from 2020 to 2030, the annual corresponding carbon sink is removed, and the predicted net carbon emissions from 2031 to 2060 are changed symmetrically, with 2030 as the axis. Considering that the carbon emission of each region in 2000 is not zero, the carbon emissions predicted from 2031 to 2060 are first standardized by 0–1, and then the extension treatment is carried out according to the value of peak carbon emission to reflect the regional carbon neutrality wish development trend under the symmetrical extension decline state from 2031 to 2060.

In scenario 2, based on the actual carbon emission level measured from 2000 to 2019 and the three carbon neutrality wish scenarios from 2020 to 2030, the annual corresponding carbon sink is removed, and the predicted net carbon emissions from 2031 to 2060 are reduced at a uniform speed to reflect the development trend of regional carbon neutrality wish under the uniform decline state scenario from 2031 to 2060.

### 4.3. Analysis of Regional Carbon Neutrality Wish

**Figure 6:**
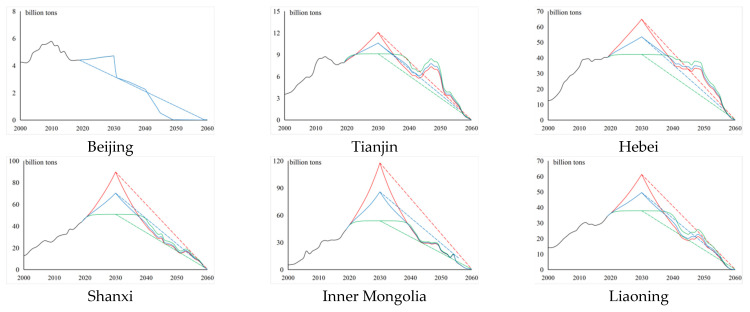

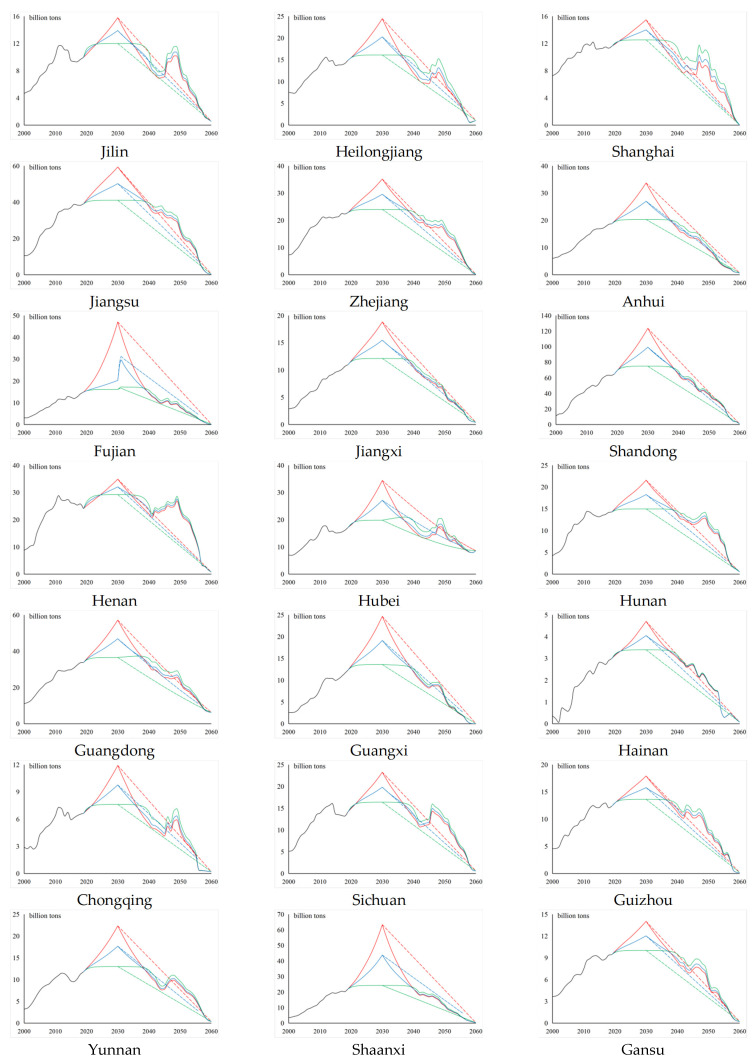

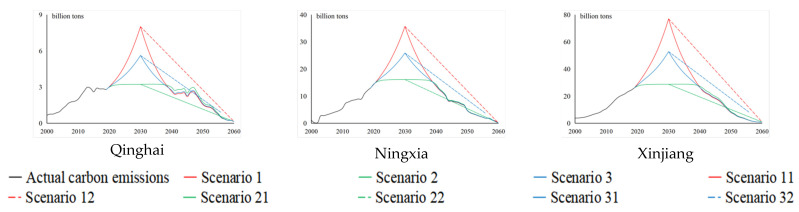
Forecast of carbon neutrality wish in various regions of China.

Based on the possible settings of carbon emissions under multifarious scenarios, we predict the carbon emissions of different regions in China from 2031 to 2060, and the results are shown in Figure 6. Owing to the substantial difference in carbon emissions among regions, the value range of vertical coordinates of each region is different in order to clearly describe the change in the trend of carbon emissions to achieve carbon neutrality wish in the regions from 2031 to 2060. In view of Beijing reaching its emission peak around 2010, its peaking path was specially treated. Under the symmetrical extension path (scenario 1), the growth rate of the symmetrical basic data is the preliminary growth rate from 2011 to 2020, based on the historical net emission data from 2000 to 2010 and using 2010 as the symmetry axis. Considering that the carbon neutrality wish year in the scenario is 2060, the horizontal stretching combination is carried out according to the change in scale, and bearing in mind the exact historical growth rate from 2010 to 2019, the change range is corrected according to the actual growth rate in that year. In the uniform decline path (scenario 2), taking the historical net carbon emission data from 2010 to 2019 and a net carbon emission value of zero in 2060 as the basic projection data, linear regression is used to combine with the fitting deviation in ordinary least squares, least absolute residuals (LAR), and Bisquare to make the best choice in comparison with the overall goodness of fit. By focusing on the 2030–2060 carbon neutral target year range, under the symmetrical extension decline state, we find that each region presents the following common characteristics. In the symmetrical extended decline state, the volatility of the ideal state is relatively large, the carbon emission level in the early stage is relatively low compared with the other two sub-scenarios, and the highest level of the three is in the average state, with its decline being the most severe in the later stage. In the uniform decline state, the carbon emissions of each region show a slow increase in the decline rate among the various states, but the decline rate of carbon emissions among regions in the same year shows few differences, while the decline rate of Guangdong is lower than that of the other provinces. The specific analysis of the six sub-scenarios is presented below.

Under the basic assumption of a symmetrical extension decline after the emission peak, in the unconstrained symmetrical extension decline state (scenario 11), the change in the trend of each region corresponds to the left-hand side of 2030. Shandong’s carbon emissions rank first from 2030 to 2056 and experience a significant decline in 2057, while simultaneously, Guangdong’s carbon emissions will be the largest. Hubei will be the largest emitting province from 2058 to 2060. In this scenario, the annual decline in China’s total carbon emissions is within [1, 7.4] billion tons and carbon emissions decrease gradually over time, with an average annual decline rate of approximately 11.82%; this rate lowers over time. In the ideal symmetrical extension decline state (scenario 21), the annual decline range of national total carbon emissions is within [0, 1.2] billion tons, but the volatility of each region is relatively large. The carbon emission level in the early stage is lower than that in the other two symmetrical extension decline state scenarios, but always at the highest level of the three in the middle stage; the decline is most intense in the later stage. The average annual decline rate is approximately 10.00%; this rate decreases with time. In the average symmetrical extension decline state (scenario 31), the carbon emission scale is always at the middle level. From 2030 to 2034, around 15 provinces will have carbon emission values greater than the overall average, and then they decline. From 2044 to 2049, around 11 provinces have carbon emission levels higher than average but eventually, only four provinces will have carbon emissions higher than the average. Overall, it can be observed that the carbon emission level will gradually converge by 2060. Please replace with the following text:

In this scenario, the annual decline in total carbon emissions in China is within [1,4] a billion tons, and the annual carbon emissions reduce gradually over time, with an average annual decline rate of approximately 11.08%. In the symmetrical extension decline state, the cumulative carbon emissions of the different scenarios from 2031 to 2060 are 1417.4, 1345.2, and 1364.2 billion tons, respectively. The carbon neutrality wish after the optimal regulated emission peak will be 53.2 billion tons less than that after the unconstrained state of emission peak, which highlights the important role of control during the emission peak period.

Under the basic assumption that carbon emissions will decline at a uniform rate after reaching the peak, the carbon emissions of each region in the unconstrained uniform decline state (scenario 12) shows a downward trend, with its rate slowly rising for all states. The total carbon emissions of the country will decline by approximately 3.85 billion tons every year, with an average annual decline rate of approximately 11.43%, and there is little difference in the decline rate of carbon emissions among the regions in the same year; especially, the decline rate of Guangdong, which is lower than that of the other provinces. In the ideal uniform decline state (scenario 22), the carbon emission of each region is similar to scenario 21, but the decline rate is the lowest under the three sub-scenarios in the ideal state. The total carbon emissions decline by approximately 2.12 billion tons per year, with an average annual decline rate of 10.74%. In the uniform decline state (scenario 32), the carbon emission of each region is similar to those in scenario 21. The total carbon emissions of the whole country decrease by approximately three billion tons every year, with an average annual decline rate of approximately 11.16%. Although the carbon emission gap under the three scenarios gradually narrowed over time and finally converged to the same carbon neutrality value, the sum of carbon emissions from 2031 to 2060 is very different due to the dissimilarities in emission peak values in the various scenarios. The cumulative carbon emissions of the three scenarios are 1729.8, 1011.5, and 1370.7 billion tons, respectively. The difficulty of regional carbon neutrality wish under diverse emission peak scenarios is not only different, but its impact on the ecological environment is not at the same level.

Based on the comprehensive analysis of carbon neutrality wish in various regions, it is easier to complete carbon neutrality wish in Hubei than in other provinces, and the carbon emission level needs to be controlled to decrease by approximately 68.22% from 2030. Nevertheless, Tianjin shows the sharpest contrast. Owing to its weak carbon sink capacity, and with the extension of human activities and further economic development, the capacity will decline. By 2060, carbon emissions need to be 0.04% of the carbon emissions of the peak year, which also poses a significant challenge to these local governments.

Under the combination of different emission peaking and neutralization scenarios, China’s overall carbon emission intensity varies greatly. The cumulative carbon emissions in the unconstrained uniform decline state (scenario 12) are the largest from 2031 to 2060, and the smallest in the ideal uniform decline state (scenario 22), with a difference of 718.3 billion tons, that is, more than six times that of the unconstrained emission peaking and nearly 11 times the peak value of ideal state. Carbon neutrality wish under multiple scenarios also proves the importance of implementing carbon emission control policies as soon as possible; otherwise, large scale carbon emissions will affect the future development of mankind.

## 5. Determinants of Regional Emission Peak and Carbon Neutrality Wish

### 5.1. Measurement Model and Data

#### 5.1.1. Brief Description of the STIRPAT Model

Using the STIRPAT model [100], this study explores the impact path of economic development level, population scale, and scientific and technological innovation on the carbon emission intensity of 30 provincial-level administrative regions of China, which is expressed as follows:

(3) $$lnI_{i}=a+blnA_{i}+clnP_{i}+flnT_{i}+e_{i}$$

where *I* is the regional energy carbon emission intensity, *A* is the regional economic development level, *P* is the regional population scale, and *T* is the regional S&T innovation capacity, while *a*, *b*, *c*, *f* are the regression coefficients of these variables, and *e_i_* is the random error term of regression.

#### 5.1.2. Estimation of Influencing Factors

According to the STIRPAT model, before exploring the impact mechanism of economic development level, population scale, and S&T innovation on regional emission peak and carbon neutrality wish, it is necessary to quantify and estimate the values of economic development level, population scale, and S&T innovation in various regions and over different periods. Since the government generally does not inhibit the progress of economic development and S&T innovation, and China has begun to encourage fertility, the population growth will not be strictly limited. Therefore, the estimation of the three influencing factors is unconstrained. The estimation method for each determinant is as follows.

Economic development level. The GDP of each region was selected to represent the regional economic development level. The actual data were obtained from the National Bureau of Statistics for 2000–2020. The forecast data consider the greater volatility of the economy. The forecast data are based on the GDP from 1992 to 2020 of the National Bureau of Statistics, and excludes 2008 (financial crisis) and 2020 (COVID-19). With the help of the “curve fitting method” in SPSS for rough projection and on the premise of passing the significance test, we selected the curve cluster with a high fitting degree and relatively close to the reality and performed arithmetic average processing. On this basis, according to the projection of China’s economic growth over different periods by the Global Energy Interconnection Development and Cooperation Organization, the predicted values of the various regions over many periods were adjusted.

Population scale. The registered residence population in each district was characterized by population size. The actual data are from the “Fifth Population Census of China (2000)” and the “Sixth Population Census of China (2010)”, which include data related to the natural population change projection, such as registered residence population and annual population growth rate. There are also data related to machinery population change projection, such as registered residence migration. Considering the large error between the 1% sampling survey data (using samples to represent the population) and the real data in each region, we filled the missing values in the middle year using the linear interpolation method. The forecast of population size consists of two parts: natural and mechanical population growth. In terms of natural population growth, considering the limited data availability at the provincial-level administrative region, we used the trend extrapolation method (at a constant growth rate) for projection. Since the population growth rate is not constant in the long-term forecast, the predicted value of the total population of each region over different periods was adjusted according to the medium plan [the total population estimated by the natural population growth rate in the plan is more in line with the projection range of major institutions and scholars in China.] in the “World Population Prospects of the 2019 Revision” issued by the United Nations. [The General Office of the State Council of the People’s Republic of China issued opinions on the furtherance of the registered residence system reform in 2014, and all provinces have generally abolished policies that promote the division of (non)-farmers.] In terms of mechanical population growth, the Markov Chain (MC) model was used to describe the dynamic changes in population growth caused by cross-provincial migration. First, the initial immigration probability vector matrix $WPMI_{1\times 31}$, initial immigration probability vector matrix $WPMO_{1\times 31}$, inter-provincial immigration probability transfer matrix $PMI_{31\times 31}$, and inter provincial immigration probability transfer matrix $PMO_{31\times 31}$ are constructed. Next, immigration probability $WI_{n}$ and immigration probability $WO_{n}$ in the n-th year can be expressed as:

(4) $$WI_{n}=WPMI\times PMI^{n-2000}$$

(5) $$WO_{n}=WPMO\times PMO^{n-2000}$$

(6) $$P_{mig\_n}=P_{mig}\times \left(WI_{n}-WO_{n}\right)$$

where *P_mig_n_* is the number of inter-provincial migrations in year *n*, *P_mig_* is the total population of inter-provincial migration, and the data are for reference. Considering the changing characteristics of China’s urbanization and in combination with the National New-type Urbanization Plan (2014–2020) issued by the State Council, China’s urbanization is expected to reach more than 70% by 2035. After the urbanization rate reaches 70%, with the equalization of public services and the realization of urban–rural integration, the inter-provincial population flow at the national level tends to become balanced. Therefore, in the mechanical projection process, the total inter-provincial population migration will not increase from 2035 to 2060.

Scientific and technological innovation. The number of patents is often used to measure the innovation level of enterprises [101] and regions [102], so the patent authorization stock of each region was selected to represent the regional S&T innovation levels. The original data was obtained from the patent authorization equivalent of the National Bureau of Statistics for 2000–2019 [103]. The perpetual inventory system was adopted to convert patent authorization data at the current time point into patent authorization stock data. Considering the rapid iteration of knowledge, the depreciation rate is 20%. Based on patent authorization stock data from 2000 to 2019, a rough projection was made with the help of the curve fitting method in SPSS. In combination with the background of encouraging innovation by the state, on the premise of passing the significance test, the curve with a growth rate greater than the average growth rate of historical data shall be preferentially selected among the curves with a high fitting degree, and arithmetic average processing shall be carried out.

**Figure 7:**
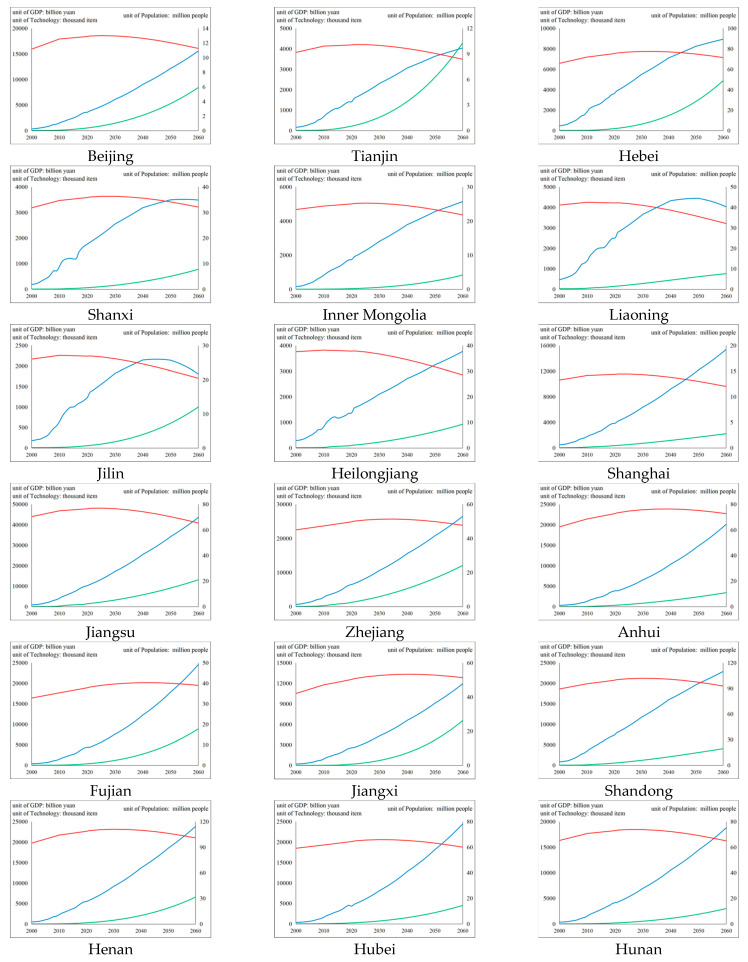

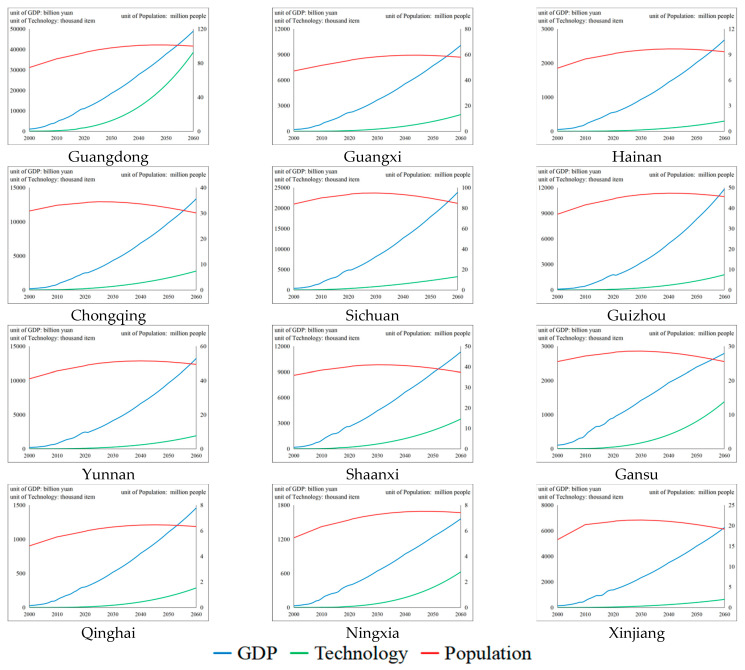
Measurement and estimation of China’s regional economic development level, population scale and S&T innovation.

The measurement and estimation results for the economic development level, population scale, and S&T innovation in different regions of China are shown in Figure 7 (the gap of the various indicators between regions are small and the value range of the vertical axis of each region are the same for purposes of comparison). The economic development level has a certain heterogeneity among regions. In 27 provinces, such as Beijing, Shanghai, and Jiangsu, there is a constant growth trend, of which Tianjin, Hebei, and Inner Mongolia show almost saturated growth, while the economic development levels of Shanxi, Liaoning, and Jilin show a declining trend over the projection period. Overall, the population scale of each region first increases and then decreases, and there are differences in the peak time points among the provinces. At present (2021), the population scales of Liaoning, Jilin, and Heilongjiang have reached their peaks. From 2021 to 2030, around 27% of provinces will reach their peak population, while the population scale of all provinces will peak before 2060. The S&T innovation in all regions shows an overall growth trend, while the annual growth rate reaches its peak from 2010 to 2021 and then decreases annually. However, the growth momentum of the different provinces in the long term is different: around half of the provinces have an average annual growth rate of 4.5–5% for S&T innovation, around 17% of the provinces are at 3.5–4.5%, and around 27% of the provinces are at 2.5–3.5%. Only Liaoning’s annual growth rate of S&T innovation is close to saturation (below 2%).

### 5.2. Empirical Analysis

A spatial panel data regression analysis was conducted with the carbon emission of each region in different scenarios and periods as the response variable and the economic development level, population scale, and S&T innovation as the explanatory variables. The Hausman test results show that the random effect model is rejected in the regression of each period, so the fixed effect model is selected for regression; the results are shown in Table 3.

Regarding the impact of economic development level in measurement periods (2000–2019), the economic development level affects carbon emissions at a significance level of 1% and the coefficient is positive, thereby indicating that the improvement in economic development level will promote the further growth of carbon emissions. For every 1 unit increase in economic level, carbon emissions will correspondingly increase by about 0.7042 units, and the three scenarios in the 2020–2030 emission peak periods differ. In scenarios 1 and 3, the economy affects carbon emissions at a significance level of 5% and the effect of economic development level on carbon emissions is weaker than in the historical periods. Each unit of economic growth will drive 0.2613 units and 0.1796 units of carbon emissions in these two scenarios, respectively. In scenario 2, economic growth will have a restraining effect of 0.0581 units on carbon emissions. This situation is due to the constraints of the ideal state scenario being very strict, such that the carbon emissions fall within the declining range of the EKC. It signifies that the improvement in the economic development level will lead to a decline in carbon emissions. In the 2031–2060 carbon neutrality wish period, the change in the carbon emissions and economic development levels extend to the end of the EKC, and compared with the uniform decline state (scenarios 12, 22, and 32), the economic development level in the symmetrical extension decline state (scenarios 11, 21, and 31) will have a stronger inhibitory effect on carbon emissions. At the 1% significance level, the inhibitory effect of economic development on carbon emission is greater than one time.

Regarding the impact of population scale over the different periods, public awareness of environmental protection is low from 2000 to 2019. The population scale promotes the rise of carbon emissions at the 1% significance level. For each unit of population scale growth, carbon emissions will increase by 4.1285 units. During the emission peak period from 2020 to 2030, the population scale will further expand and form a population scale effect, that is, when the population scale reaches a certain value, the consumption of unit resources will be reduced. Simultaneously, given the augmented environmental protection awareness and the continuous improvement in population quality consequent to the higher education level, there will be a decoupling of the population scale and carbon emissions at this stage. In the carbon neutrality wish period from 2031 to 2060, the mechanical change in population scale in various regions tends to be stable and the annual natural growth rate is negative, which results in a greatly weakened population scale effect. In each scenario, the population scale will promote the increase in carbon emissions at a significance level of 1%, and one unit of population growth will bring 11.8982 (scenario 11), 11.0882 (scenario 12), 13.2984 (scenario 21) and 10.5214, respectively (scenario 22), 12.4422 (scenario 31) and 10.8326 (scenario 32) of carbon emission growth.

Considering the time lag between the output of S&T innovation and their industrial application, the impact of S&T innovation on carbon emissions will show an obvious heterogeneity over the three periods. From 2000 to 2019, the progress of S&T has made social development more intelligent and reduced human participation in production activities to a certain extent, which will inhibit the growth of carbon emissions at the 1% significance level. Every unit of S&T innovation capacity will inhibit carbon emissions by 0.2977 units. The main technology application in the emission peak stage from 2020 to 2030 is the non-green innovation output during the rapid economic development in the early stage, which accelerates the growth of carbon emissions at the 1% significance level, and from 2031 to 2060, the main technology application in the carbon neutrality wish stage is the green innovation output in the emission peak stage. Except under scenario 21, which inhibits 0.4274 units of carbon emissions per unit of S&T innovation at the 5% significance level, the other five scenarios inhibit the growth of carbon emissions at the 1% significance level, and its effect is about twice that of the S&T innovation in curbing human activities. Therefore, improving the output of green innovation is a relevant factor in helping China’s emission reduction.

## 6. Discussion

### 6.1. Conclusions

Global climate change caused by CO_2_ and other greenhouse gas emissions has become one of the biggest challenges of this century. China has gradually embarked on a new path of high-quality development, guided by ecological priority and green development, and put forward the major commitment of “striving to reach the peak of carbon emissions by 2030 and strive to achieve carbon neutrality by 2060”. However, as the largest carbon emitter, the process of achieving its emission peak and carbon neutrality wish is worth delving into. To explore the low-carbon development path in the current period and emission peak and carbon neutrality wish periods from the land use perspective, this study considers emission peak and carbon neutrality wish as its objectives, and constructs six carbon emission paths from 2000 to 2060 by setting three emission peak scenarios—unconstrained state scenario, ideal state scenario, and average state scenario—and two carbon neutrality wish scenarios of symmetrical extension decline state scenario and uniform decline state scenario. To predict the emission peak stage, based on the final energy consumption data of the different provinces in China from 2000 to 2019, this study estimates future carbon emissions by simulating and predicting its growth rate. For the projection of the carbon neutrality stage, the projection of carbon sequestration is based on 1-km land use grid data with an interval of five years from 2000 to 2020. Based on actual regional carbon emissions from 2000 to 2018 and the carbon emission projection value under three scenarios from 2019 to 2030, the future carbon emission value is estimated by predicting the scale of net carbon emissions. To explore the impact mechanism of carbon emissions in different periods, this study uses the STIRPAT model, based on panel data of 30 provincial-level administrative regions from 2000 to 2060, and takes carbon emissions as the response variable, and economic development level, population scale, and S&T innovation as the explanatory variables, to explore the impact of various factors that influence the emission peak and carbon neutrality wish path.

In achieving the emission peak in an unconstrained state (scenario 1), the total carbon emissions of all regions in China will increase from 645.74 billion tons (2020) to 1175.24 billion tons (2030), of which Shandong has the highest carbon emission intensity among all regions, while Hainan and Qinghai have the lowest. At the same time, the heterogeneity of carbon emissions among regions will increase year by year, and the standard deviation in carbon emissions in all provinces will expand from 15.57 billion tons (2020) to 30.89 billion tons (2030). Six provinces, including Inner Mongolia, will become the six regions with the fastest carbon emissions growth if they do not exercise control. Under this scenario, all regions in China will emit 9749.00 billion tons of carbon from 2020 to 2030. In the ideal state scenario (scenario 2), the total carbon emissions of all regions in China will increase from 636.90 billion tons (2020) to 675.15 billion tons (2030), and the development trend of carbon emissions among regions will converge, resulting in the standard deviation in carbon emissions in all provinces only expanding from 15.37 billion tons (2020) to 16.46 billion tons (2030). In this scenario, the total carbon emissions of all regions in China from 2020 to 2030 will be 7349.31 billion tons, which is 2399.69 billion tons less compared to the unconstrained state, indicating that the effect of decarbonization is significant. In the average state scenario (scenario 3), the total carbon emissions of all regions in China will increase from 639.10 billion tons (2020) to 922.83 billion tons (2030), but the difference between various regions and their maximum/minimum carbon emissions is still large (94.34%). The standard deviation in carbon emissions of all provinces will only expand from 15.43 billion tons (2020) to 23.30 billion tons (2030). In this scenario, China’s regional carbon emissions from 2020 to 2030 will total 8523.97 billion tons, an increase of 1174.66 billion tons compared with the ideal state, and a decrease of 1225.03 billion tons compared with the unconstrained state. The local government should make reasonable decisions based on the specific development of the region since the total carbon emissions will vary greatly under different carbon peak paths.

If realizing carbon neutrality in a state of unconstrained symmetrical extension decline (scenario 11), the annual decline in China’s total carbon emissions will be around [1, 7.4] billion tons with an average annual decline rate of about 11.82%, thus showing a gradual decline over time. Under this scenario, the cumulative carbon emissions from 2031-2060 will be around 14208.54 billion tons. In the ideal symmetrical extension decline state (scenario 21), the annual decline in China’s total carbon emissions will be around [0, 1.2] billion tons, albeit each region will see significant volatility, with an average annual decline rate of 10.00% and a gradual decline over time. Under this scenario, the cumulative carbon emissions from 2031–2060 will total 13,486.68 billion tons. In the average symmetrical extension decline state (scenario 22), the annual decline in China’s total carbon emissions will be around [1,4] billion tons with an average annual decline rate of approximately 11.08%. The carbon emissions level will gradually converge by 2060. In this scenario, the cumulative carbon emissions from 2031–2060 will total 13,675.87 billion tons. In the unconstrained uniform decline state (scenario 12), China’s total carbon emissions will decline by approximately 3.85 billion tons per year, with an average annual decline rate of approximately 11.43%. The carbon emissions in the various regions will register a slow increase in the decline rate. For the decline rate among regions in the same year, there is little difference among provinces, except for Guangdong. In this scenario, the cumulative carbon emissions from 2031–2060 are 17,343.76 billion tons. In the ideal uniform decline state (scenario 22), China’s total carbon emissions will decline by approximately 2.12 billion tons per year, with an average annual decline rate of 10.74%. Under this scenario, the cumulative carbon emissions from 2031–2060 will total 10,160.80 billion tons. Under the average uniform decline state (scenario 32), China’s total annual decline is around three billion tons, with an average annual decline rate of approximately 11.16%. In this scenario, the cumulative carbon emissions from 2031–2060 will total 13,752.28 billion tons. Although the final carbon neutrality goal is the same in the six cases, there are obvious variations in the degree of environmental pollution in the different situations, resulting in a huge difference in the total carbon emissions in the carbon neutral phase: the carbon emissions in the ideal uniform decline state (Scenario 22) can be reduced by up to 7182.96 billion tons compared with the unconstrained uniform decline state (Scenario 12), and the minimum difference in carbon emissions in different states is also 76.41 billion tons.

In light of the research on the pathways affecting carbon emissions from 2000 to 2019, the impact of economic development level, population scale, and S&T innovation on carbon emissions is consistent with the actual situation. This fact verifies that the STIRPAT model is reasonable and can be used to further explore the impact of the three influencing factors on carbon emissions in divergent scenarios. In the 2020–2030 carbon emission peak period, the economic level and carbon emissions under the optimal policy effect will fall within the declining range of the EKC, while the other two scenarios are in the growth range. Population development will be influenced by both the population scale effect and the popularization of environmental protection awareness, thus resulting in a decoupling phenomenon. For S&T innovation, the industrial application of S&T innovation is the non-green innovation produced in the period of rapid economic development in the previous stage, which will lead to a further increase in carbon emissions. In the carbon neutrality period from 2031 to 2060, with the development of China’s economy and the implementation of emission reduction projects, the economic development level and carbon emissions will always fall within the declining range of the EKC, while the population scale will also decline to a certain extent at this stage, thus leading to the increase in carbon emissions. For S&T innovation, the industrial application of innovative technologies in the pre carbon-neutrality period is mainly based on the green innovation output for the emission peak period, which is essential to achieving carbon neutrality. In short, in the emission peak phase, the role of the economy and S&T in emission reduction depends on the choice of different emission peak paths, while population is decoupled from carbon emissions. In the pre-carbon neutrality phase, no matter the pathway, the economy and S&T will both play an active role in the emission reduction process.

### 6.2. Marginal Contributions

First, focusing on the development goal of reaching the emission peak by 2030, this study considers three scenarios under the unconstrained, ideal, and average state scenarios, which not only measures the possible range of the emission peak, but also estimates the most likely peak. Looking at the development prospects for carbon neutrality wish by 2060, this study further simulates the symmetrical extension decline state and uniform decline state, and constructs six combined scenarios of carbon emissions from 2000 to 2060 to predict the development path of emission peak and carbon neutrality wish in different regions and diverse scenarios.

Second, there may be large errors in the projections based on China’s total carbon emissions. In this study, carbon emissions were divided into three different periods according to measurement and projection. By predicting the carbon emission scale of provincial-level administrative regions and summing them up, we can obtain the overall carbon emissions of each time node. The carbon emissions of each region are small, meaning absolute errors are also small, which can reduce the overall error to a certain extent. Nevertheless, the estimation error of carbon emissions among regions can offset and reduce the overall error to a certain extent.

Third, this study estimates not only the emission peak in 2030 and carbon neutrality wish in 2060 but also the economic development level, population scale, and scientific and technological innovation of each region. It further examines their impact on carbon emissions in different scenarios based on the STIRPAT model to explore the road of low-carbon development in China.

Moreover, under the setting of various situations, the regional emission peak can seemingly be achieved instantaneously in 2030 in this paper theoretically. Yet in reality, such a change is difficult and improbable. Although many regions are striving to achieve carbon peaking in 2030 based on the emission peak target set by China, this only provides them with a clear deadline for reaching the emission peak. Based on this time point, this paper tries to set various theoretical scenarios and analyze the possible timelines of emission peaks in different regions based on the latest data of regional emission peaks. Nevertheless, combined with the target of carbon neutrality from 2030 to 2060, the regional emission peak in 2030 can still be divided into an ideal (emission peak with decreasing growth rate of carbon emissions) and non-ideal situation (unconstrained emission peak). If the emission peak is achieved in an ideal situation, the carbon neutrality pressure faced by the region in the future will be reduced, and the accumulated carbon emissions in the future will also be greatly reduced. In a non-ideal situation, the region will face the opposite situation. This paper also estimates the role that the economy, population, and S&T will play in different scenarios. Although these scenarios are theoretical, they can still provide local governments with insights for policy making. In addition, if the region has achieved emission peaking before 2030, by comparing the situation then to the various scenarios laid out in this paper, we can further clarify the contribution of the region in reducing total carbon emissions.

### 6.3. Policy Suggestions

Based on the scenario simulation of emission peak and carbon neutrality wish, this study puts forward the following policy suggestions as a reference for local governments to prepare reasonable emission reduction plans. First, we formulate a systematic long-term emission reduction strategic layout and implement regionally differentiated decarbonization policies. We should pay attention to not only short-term emission reduction targets, but also long-term carbon emissions control performance, especially to strengthen the implementation of emission reduction policies in Inner Mongolia, Fujian, Shaanxi, Qinghai, Ningxia, and Xinjiang, and control the growth rate of carbon emissions and smoothly complete the task of reaching the peak in 2030. Second, we should flexibly adjust emissions reduction policies according to the temporal and spatial patterns of carbon emissions. We should monitor the dynamic changes in carbon emissions in provincial-level administrative regions, thus maintaining the overall balance of emission reduction policies, especially the balance between the optimal-controlled area (Hainan, Qinghai) and the worst-controlled area (Shandong), and realizing the optimal combination of overall policy and regional policy. Third, we must avoid blindly pursuing the carbon neutrality target of 2060 and ignoring the destructive effect of cumulative carbon emissions on the environment. On the premise of taking 2060 as the carbon neutrality target year, all regions should explore their optimal regional carbon neutrality wish paths based on the emission reduction principle of scientific decarbonization and seeking stability reduction.

Based on the empirical analysis of the impact path of emission peak and carbon neutrality wish, this study holds that, first, the impact of economic development on carbon emissions falls within the declining range of the EKC, which is key to realizing carbon neutrality wish. According to the “Blue Book of China’s Society” issued by the Chinese Academy of Social Sciences, China will become a high-income country in the 14th Five-Year Plan period. China must thus improve energy efficiency, develop renewable energy resources, optimize energy consumption structure, enhance industrial structure, and accelerate the transformation from a high energy consumption development mode to a low-carbon green development mode. Second, the population density of the control area should be in an adequate range to prevent the reduction in the population scale effect due to an extremely small population density or the enhancement of migration activities following an extremely large population density, thus resulting in an increase in carbon emissions. In addition, the relevant departments should promote clean energy, low-carbon lifestyle, and quality education to the majority of residents to reduce household carbon emissions. Third, environmentally friendly S&T innovation is the main driving force for carbon emission reduction. To realize the gradual decline in carbon emissions, we should strengthen the research and development and the industrial applications of low-carbon technologies, such as carbon capture technology. Additionally, the Chinese Central Government should improve the corresponding system construction, such as establishing a perfect carbon trading market to force high-energy-consuming enterprises to reduce energy consumption and improve energy output efficiency.

### 6.4. Limitations and Scope for Future Research

First, this study assumes an ideal state in the scenario simulation. It does not consider the upheavals in natural factors, such as major disasters, the impact of human factors, including conflict and war, the basic constraints of revolutionary S&T innovation, and the application of new energy. In reality, there are many uncontrollable external factors that interfere with the operation of carbon emission systems, such as COVID-19. In future, we will attempt to build a System Dynamic model of the emission reduction system by taking into consideration a variety of external factors and provide more realistic conclusions and suggestions.

Second, the scenario set in this study is based on a theoretical economic trend, meaning the actual future carbon emission trend will have a certain random deviation from the scenarios contemplated in this study. In the future, we will consider a multi-dimensional situation and introduce disturbance factors to build a more comprehensive simulation.

Third, the data to measure historical carbon emissions are based on the “China Energy Statistics Yearbook”. There are practical problems such as omissions, which produce a gap in information and will have a certain impact on follow-up research. We should thus consider using more accurate and complete carbon emissions data in future.

## Figures and Tables

**Table 1:** The regression results of the Tobit model.

Land Use Status Classification		Region	Coefficient of Carbon Sinks	References
Category	Type			
Woodland	Closed forest land	Nationwide	0.87 $t\cdot {\mathrm{hm}}^{-2}\cdot a^{-1}$	[93,94]
	Shrubbery		0.23 $t\cdot {\mathrm{hm}}^{-2}\cdot a^{-1}$	
	Sparse wood land		0.58 $t\cdot {\mathrm{hm}}^{-2}\cdot a^{-1}$	
	Other woodland		0.2327 $t\cdot {\mathrm{hm}}^{-2}\cdot a^{-1}$	
Grassland	High-coverage grassland	Nationwide	0.138 $t\cdot {\mathrm{hm}}^{-2}\cdot a^{-1}$	[95,96]
	Medium-coverage grassland		0.046 $t\cdot {\mathrm{hm}}^{-2}\cdot a^{-1}$	
	Low-coverage grassland		0.021 $t\cdot {\mathrm{hm}}^{-2}\cdot a^{-1}$	
Waters	Canals	Nationwide	0.671 $t\cdot {\mathrm{hm}}^{-2}\cdot a^{-1}$	[97]
	Lakes		0.303 $t\cdot {\mathrm{hm}}^{-2}\cdot a^{-1}$	
	Reservoir and pond		0.303 $t\cdot {\mathrm{hm}}^{-2}\cdot a^{-1}$	
	Beach land		0.567 $t\cdot {\mathrm{hm}}^{-2}\cdot a^{-1}$	
	Shiedles		0.567 $t\cdot {\mathrm{hm}}^{-2}\cdot a^{-1}$	
Unused land		Nationwide	0.0005 $t\cdot {\mathrm{hm}}^{-2}\cdot a^{-1}$	[98]
Cultivated land		Northeast China	5.23 $t\cdot {\mathrm{hm}}^{-2}\cdot a^{-1}$	[99]
		East China	7.04 $t\cdot {\mathrm{hm}}^{-2}\cdot a^{-1}$	
		Central China	7.61 $t\cdot {\mathrm{hm}}^{-2}\cdot a^{-1}$	
		West China	4.23 $t\cdot {\mathrm{hm}}^{-2}\cdot a^{-1}$	

**Table 2:** Meaning of various scenario.

Scenario	Meaning
Scenario 11	Symmetrical extended decline state of carbon neutrality wish, after unconstrained state of emission peak
Scenario 12	Uniform decline state of carbon neutrality wish, after unconstrained state of emission peak
Scenario 21	Symmetrical extended decline state of carbon neutrality wish, after ideal state of emission peak
Scenario 22	Uniform decline state of carbon neutrality wish, after ideal state of emission peak
Scenario 31	Symmetrical extended decline state of carbon neutrality wish, after average state of emission peak
Scenario 32	Uniform decline state of carbon neutrality wish, after average state of emission peak

**Table 3:** Regression results of influencing factors of China’s regional carbon emissions in different periods.

	2000–2019	2020–2030			2031–2060					
	Measure	Scenario 1	Scenario 2	Scenario 3	Scenario 11	Scenario 12	Scenario 21	Scenario 22	Scenario 31	Scenario 32
GDP	0.7042 ***	0.2613 **	−0.0581 **	0.1796 ***	−1.1038 ***	−0.9047 ***	−1.0701 ***	−0.8074 ***	−1.0807 ***	−0.8654 ***
	(0.000)	(0.047)	(0.007)	(0.029)	(0.000)	(0.000)	(0.000)	(0.000)	(0.000)	(0.000)
Population	4.1285 ***	0.7004	0.1421	0.3449	11.8982 ***	11.0882 ***	13.2984 ***	10.5214 ***	12.4422 ***	10.8326 ***
	(0.000)	(0.219)	(0.129)	(0.336)	(0.000)	(0.000)	(0.000)	(0.000)	(0.000)	(0.000)
Technology	−0.2977 ***	0.3780 ***	0.0649 ***	0.2296 ***	−0.7022 ***	−0.6337 ***	−0.4274 **	−0.6161 ***	−0.5875 ***	−0.6279 ***
	(0.000)	(0.000)	(0.000)	(0.000)	(0.000)	(0.000)	(0.013)	(0.000)	(0.000)	(0.000)
_cons	−72.6320 ***	−16.5380 ***	0.2083	−7.7037	−182.7072 ***	−171.5302 ***	−211.2435 ***	−163.4547 ***	−194.0237 ***	−167.7973 ***
	(0.000)	(0.000)	(0.897)	(0.214)	(0.000)	(0.000)	(0.000)	(0.000)	(0.000)	(0.000)
rho	0.9946	0.9944	0.9997	0.9950	0.9916	0.9938	0.9930	0.9943	0.9922	0.9940
R2	0.7985	0.8500	0.5841	0.8501	0.6560	0.6925	0.5805	0.7123	0.6245	0.7016

Note: P statistics in brackets; *** and ** represent significant at the levels of 1% and 5%, respectively. The _cons represents a constant term, which is the intercept term of the regression equation. The rho represents the percentage of individual effects in the total error term and goodness of fit, while the larger the rho, the more errors come from individuals and the more support for using fixed effects model. The R2 represents goodness of fit, which is an important criterion to judge whether a model fits well or not. The larger the R2, the better the model fits.

## Data Availability

No new data were created or analyzed in this study. Data sharing is not applicable to this article.
